# An MPTCP-Based Transmission Scheme for Improving the Control Stability of Unmanned Aerial Vehicles

**DOI:** 10.3390/s21082791

**Published:** 2021-04-15

**Authors:** Woonghee Lee, Joon Yeop Lee, Hyeontae Joo, Hwangnam Kim

**Affiliations:** School of Electrical Engineering, Korea University, Seoul 02841, Korea; tgorevenge@korea.ac.kr (W.L.); charon7@korea.ac.kr (J.Y.L.); motern800@korea.ac.kr (H.J.)

**Keywords:** UAV, Multipath Transmission Control Protocol, reliable transmission

## Abstract

Recently, unmanned aerial vehicles (UAVs) have been applied to various applications. In order to perform repetitive and accurate tasks with a UAV, it is more efficient for the operator to perform the tasks through an integrated management program rather than controlling the UAVs one by one through a controller. In this environment, control packets must be reliably delivered to the UAV to perform missions stably. However, wireless communication is at risk of packet loss or packet delay. Typical network communications can respond to situations in which packets are lost by retransmitting lost packets. However, in the case of UAV control, delay due to retransmission is fatal, so control packet loss and delay should not occur. As UAVs move quickly, there is a high risk of accidents if control packets are lost or delayed. In order to stably control a UAV by transmitting control messages, we propose a control packet transmission scheme, *ConClone*. *ConClone* replicates control packets and then transmits them over multiple network connections to increase the probability of successful control packet transmission. We implemented *ConClone* using real equipment, and we verified its performance through experiments and theoretical analysis.

## 1. Introduction

Recently, unmanned aerial vehicles (UAVs) have been used for various studies and services. Additionally, service providers are looking for ways to apply UAVs with high moving speed and three dimensional movement to various tasks [1,2,3]. A UAV can be equipped with a small computing board, which enables a variety of tasks, such as collaboration with existing applications [4], rapid object delivery [5] or a wide range of information gathering or sharing tasks [6,7]. UAVs for specific tasks are often managed by a ground control system (GCS) rather than being controlled by each operator one by one [8,9]. In order for the GCS to control the UAV, it must transmit its current status to the GCS, and the GCS controls the UAV maneuvers by transmitting control packets to it. However, wireless networks for controlling UAVs are likely to lose or delay control packets due to unstable network environments. As the UAV moves with very high speed, delayed transmission of control packets can lead to fatal accidents. Therefore, transmission of control packets must be fast and reliable. In addition, since it is important that control messages arrive within the time limit, the environment for transmitting control messages should be in the domain of a Time Sensitive Network (TSN). Based on these observations, we propose *ConClone*, a scheme for control packet transmission in order to reliably transmit control packets to the UAV over TSN.

*ConClone* is based on the Multipath Transmission Control Protocol (MPTCP), which can maintain multiple connections simultaneously through multiple interfaces [10]. Multiple interfaces used in MPTCP can use different frequency bands or combine different interfaces (e.g., UWB + Wi-Fi, LTE + Bluetooth and wireless + wired) to avoid channel collisions or interference. MPTCP can continue to communicate through other connections, even if the communication environment of some connections is degraded. However, MPTCP cannot prevent already transmitted packets from being delayed or lost. To solve these problems, *ConClone* sends replicated control packets to the UAV through different connections. The UAV exchanges with the GCS various data such as position information [11], captured videos [12], sensor information [13] or information of nearby UAV during maneuver [14]. Replication of all packets causes a large amount of unnecessary transmissions, but *ConClone* reduces the burden on the network by selectively replicating control packets only. With *ConClone*, we can increase the reliability of UAV control by increasing the control packet delivery rate. The improvement of the control packet transmission rate, as a result, increases the probability that the control packet will arrive in time. It is possible to implement a TSN for UAVs through *ConClone*, increasing the stability of the UAV control. We implemented *ConClone* on real devices and proved its performance through experiments. Moreover, we theoretically analyzed *ConClone* to demonstrate the performance of *ConClone*.

The main contributions of our paper are summarized as follows:

- To increase the maneuver reliability of UAV, *ConClone* reduced the loss rate of control packets by transmitting replicated control packets through multiple connections.
- *ConClone* replicates only control packets to minimize the overhead of the network.
- *ConClone* has been implemented on a real device and has proven its effective performance through experiments.

This paper is organized as follows. The related work is described in Section 2. Section 3 describes the concept of *ConClone*. Section 4 analyzes the expected performance of *ConClone* theoretically. Section 5 describes the experimental setup and the performance evaluation of *ConClone*. Section 6 concludes the paper.

## 2. Related Work

This section describes studies similar to the proposed *ConClone*. There are some studies to reduce the transmission delay by modifying the communication scheme in the transport layer or the application layer. Dudek et al. proposed a system to reduce the delay by disabling Nagle’s Algorithm [15] for robot control systems [16]. Nagle’s algorithm collects data packets in a buffer instead of immediately transmitting them. When a certain amount of data are collected or an acknowledgement is received, the data are bound in packets and transmitted. Nagle’s algorithm has the advantage of increasing the efficiency of the network, but it has the disadvantage of increasing the delay. However, disabling Nagle’s algorithm increases the network congestion, which could cause overall throughput degradation. There are several studies to increase the stability of wireless network against packet loss or delay through feedback control in networked control systems (NCS) [17,18,19]. However, they cannot be directly applied to TCP, especially MPTCP, since their feedback control scheme should be embedded into the TCP of the network protocol stack within the operating system, which is not easy, and it should be allowed to access physical layer information, which is forbidden in the layered network protocol architecture. Nie et al. suggested an improved packet retransmission scheme [20]. This scheme was based on a multi-hop efficient transmission network platform composed of multiple wireless nodes. When the server received multiple data sets, this scheme suggested sending the number of lost data packets in a single ACK. This reduction in the number of ACKs had the effect of reducing traffic in a multi-hop environment. Rana et al. suggested a method to increase transmission reliability through multiple transmission attempts in UAV communications [21,22]. In this paper, fault analysis was carried out and it was suggested that 11 attempts were required for a 99% success rate of data transmission. However, if there are 11 redundant transmissions in a typical single-path communication, the overall throughput would be inevitably reduced. As a result, this paper suggested that it is very difficult to maintain high reliability in a single-path communication. In addition, a variety of studies have been conducted to cope with packet loss, such as switching techniques [23], building multiple sub-systems for reliable transmission [24,25], and analyzing packet loss rates [26,27]. In the physical layer, transmission errors could be detected using a channel coding scheme [28] or a hybrid automatic repeat request (HARQ) [29].

There are studies to improve the packet transmission reliability of control packets using MPTCP. Sayit et al. introduced various MPTCP schedulers [30]. In order to utilize multiple paths, the scheduler that could allocate packets to transmission path has been developed in various ways. The authors analyzed the reliability of MPTCP by analyzing nine different schedulers. In addition, Mondal et al. proposed an MPTCP scheduler that could allocate a dedicated connection to the control packet and could make changes to another sub-socket when there was a problem with the primary connection [31]. Additionally, Rao et al. proposed to use MPTCP for wireless communications of robots/UAVs [32]. They compared links using different MPTCP algorithms (BALIA, LIA, OLIA and WVEGAS) to links in a single TCP and demonstrated that MPTCP could provide improved stability in the conductivity of robots/UAVs. However, although these schemes could guarantee the throughput of control packets, data packets would have fewer available connections than the existing MPTCP. Therefore, these schemes are not able to fully utilize multiple connections, which are the advantages of MPTCP. Jung et al. proposed a scheduler that could distribute data to each connection based on response times, including computing, transmission and I/O times [33]. The proposed scheduler could guarantee throughput due to high bandwidth usage. However, instead of high utilization of multiple connections, the proposed scheduler could not guarantee reliable transmission of specific packets (e.g., control packets). Vu et al. suggested redundant MPTCP schedulers for desired packet latency. They estimated the latency of TCP connection and used replicated packets to provide reliable data transmission [34]. The scheme they proposed can be effective in networks that require large amounts of data transmission. However, it is not possible to fully guarantee the robustness of the control packet transmission in the limited data capacity of the wireless connection, because the priority is not considered for the control packet.

Compared to related work, *ConClone* has advantages in various aspects. *ConClone* is based on MPTCP’s default scheduler, so the throughput is not degraded. In addition, *ConClone* minimizes the network burden by replicating only the control packets, which occur far less frequently than normal data packets. Overall, *ConClone* can maximize the throughput of data packets using MPTCP while minimizing the delay and loss that can occur in control packet transmissions.

## 3. ConClone

This section describes the *ConClone* concept we implemented. Additionally, we describe in detail how *ConClone* works in the environment where a GCS remotely controls a UAV.

### 3.1. Packet Replication

The control packet contains the control information necessary for maneuvering the UAV. Considering the movement speed of the UAV, the control packet should reach the UAV without being lost or delayed. For this purpose, when the control packet is replicated and transmitted, the delivery probability of the control packet can be increased. However, it is difficult to cope with the delay of a packet only through the technique of simply replicating. Therefore, *ConClone* uses the multipath routing technique described in Section 3.2.

### 3.2. Packet Transmission through Multiple Connections

*ConClone* is based on MPTCP. With MPTCP, data packets can be delivered to the receiver through multiple connections. However, when the network environment of a specific connection deteriorates, packets with deteriorated connections are delayed, as in normal network communication. The existing MPTCP is difficult to prevent data delay caused by changes in the network environment due to its reactive behavior. Even if the packet replication method shown in Section 3.1 is used, if the network environment of the connection transmitting redundant data is deteriorated, all redundant data are delayed. This problem occurs in the MPTCP default scheduler. The default scheduler selects a connection for packet transmissions based on Smoothed Round Trip Time (SRTT). This scheduler sends more packets over better connections and the overall throughput of packet transmissions is high. However, since it takes time to detect the performance of each connection and re-select the connection, replicated packets are likely to be transmitted through a single connection rather than various connections. With a round-robin scheduler, a replicated packet can always be transmitted through another connection. However, because the round-robin scheduler does not consider the quality of the connection, the overall throughput is lower than the default scheduler based on SRTT.

**Figure 1:**
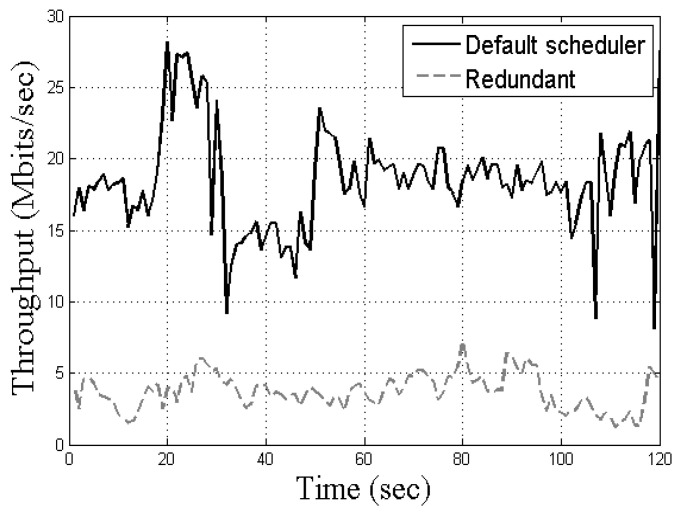
Throughput comparison of Multipath Transmission Control Protocol (MPTCP) schedulers.

Like *ConClone*, there is a redundant scheduler that replicates data to increase the probability of receiving data. The MPTCP’s redundant scheduler transfers all packets by replicating them equally to multiple paths. With a redundant scheduler, control packets are replicated to each transmission path, hence the success rate of transmitting control packets. However, because the redundant scheduler replicates both the control packet and the data packet, total throughput is lower than the default scheduler of MPTCP. To show the performance difference between the default scheduler and redundant scheduler, we experimented on actual MPTCP transmission in the experimental environment with two transmission paths. Figure 1 shows the throughput difference between the default scheduler and the redundant scheduler. The overall size of the data packet, including video or collected sensor information, is bigger than the control packet containing moving coordinates or commands. However, the redundant scheduler replicates and transmits not only control packets but also data packets. Therefore, the redundant scheduler is less efficient than the default scheduler when transferring packets, which can reduce the transmission speed of the control packets.

To solve these problems, *ConClone* transmits general data packets based on a default MPTCP scheduler, which guarantees throughput. Additionally, the replicated control packet is distributed to all the connections by *ConClone*. With *ConClone*, at least one control packet arrives at the receiver without delay, unless the transmission performance of all connections is deteriorated. *ConClone* is the scheme for preventing the loss and delay of control packets by transmitting replicated control packets through multiple connections. Thus, it is possible to achieve a high transmission success rate for control packets while ensuring high throughput of data packets.

### 3.3. Design of *ConClone*

**Figure 2:**
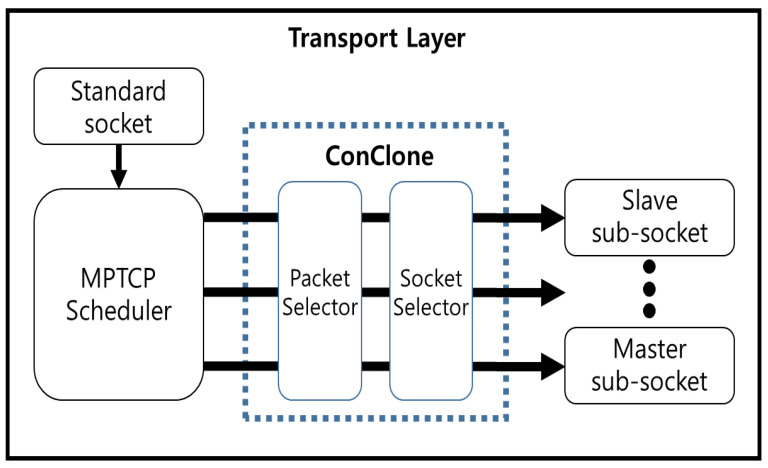
The design of *ConClone*.

MPTCP is implemented in the transport layer. The data packet generated in the application layer is transmitted to the transport layer, and the transmitted data packets are distributed through the MPTCP scheduler to a sub-socket that manages each connection. As shown in Figure 2, *ConClone* is implemented between the MPTCP scheduler and the sub-socket. *ConClone* analyzes packets to find the control packet and applies the *ConClone* scheme to the control packet only. Additionally, as shown in Figure 2, *ConClone* consists of a packet selector and a socket selector.

#### 3.3.1. Packet Selector

The *packet selector* analyzes packets to find out control packets and replicate them. MPTCP and socket buffer related functions are implemented in the Linux kernel layer. sk_buff in the kernel contains information about the buffer [35], and the *packet selector* can access sk_buff to check which packet is a control packet. Control packets are replicated by the *packet selector* as many times as the number of sub-sockets and are sent to the *socket selector*.

If the application classifies control packets separately, *packet selector* can replicate the control packet immediately at the application layer. This is because *packet selector* already knows which one is a control packet. In this case, *packet selector* ignores the process of checking the packet through sk_buff.

#### 3.3.2. Socket Selector

The *socket selector* assigns each control packet replicated in the *packet selector* to each sub-socket. The order of selecting sub-sockets is the same as the order of sub-sockets selected in the existing MPTCP scheduler. Since the MPTCP scheduler has information on the performance of the sub-socket, the *socket selector* allocates the control packet to the sub-socket in the order of performance and transmits them.

Overall, through the process of the *packet selector* and the *socket selector*, control packets are replicated by the number of sub-sockets, and control packets can be transmitted through all sub-sockets. If the receiver receives multiple packets with the same sequence number, the receiver ignores the replicated packets except the first packet received. Therefore, replicated packet transmission does not cause any problems.

## 4. Analysis of *ConClone*

In this section, we analyze the performance of *ConClone* through theoretical analysis. We investigate network problems that may occur in general network communication, and see if we can reduce the incidence of problems by using *ConClone* with multiple connections.

### 4.1. Analysis of the Network Problem Situation

In a UAV network, when data should be transmitted, data are usually transmitted through the Transmission Control Protocol (TCP), which is a reliable transmission protocol. Transmission through the TCP can be divided into three phases [36]. In the stable phase, there is no problem with transmission. The congestion phase presents the case that data transmission is delayed due to excessive data transmission or packet loss occurs due to buffer overflow. If a packet is lost, TCP starts to retransmit the lost packet. However, even if retransmission is conducted instantaneously, a UAV will receive the control packet with an additional delay of at least one round trip time (RTT). The disconnected phase includes the connection lost, retransmission timeout (RTO) and 3 duplicated ACKs, in which data transmission is greatly delayed or failed. These phenomena occur in situations where continuous transmission is not possible. In these cases, a transmitter should wait a long time for the problem to be solved or immediately establish another connection. The disconnected phase is uncommon in wired networks, but it is often seen in the case of wireless access networks, multi-hop communications and mobile communications. Since most UAVs use Wi-Fi 2.4GHz ISM band for data transmissions, the disconnected phase frequently occurs. Additionally, in the environment where the network is composed with moving objects such as UAV or mobile devices, data transmission path changes occur frequently, and the network signal strength is very unstable. In this case, data transmissions are delayed or network connections are disconnected frequently. When the operator controls a UAV, the congestion phase and the disconnected phase should occur as little as possible. Therefore, in order to prevent the congestion and disconnected phase, *ConClone* proactively responds to the phases by using multiple connections.

### 4.2. Analysis of ConClone

Prior to the actual experiment, we analyzed how much *ConClone* improves the transmission success rate of the control packet. Based on the system reliability theory [33], we analyzed the time required for transmission of control packets and the effect of multiple interfaces. Various failures could occur during transmission of control packets, such as unintended packet losses or them not arriving on time. Note that this analysis cannot be affected by the underlying physical layer. This is because TCP can see only the remaining network bandwidth after being consumed or wasted by the underlying protocol layers, such as the network, data-link and physical layers. Another reason is that TCP cannot directly see the transmission failures or bandwidth changes due to various effects in physical layer, but it perceives those failures or changes only with TCP delay or lost packets. It means this analysis can be done, independently of any physical layer assumption or modeling.

The failure of the replicated control packet on the *i*-th interface is defined as $f_{i}$. In accordance with this definition, in Section 4.2.1 we modeled on the probability of successful transmission when using *n* multiple interfaces to determine both the ideal and realistic transmission times with *ConClone*. We also derived how the transmission time varies with the number of interfaces. In Section 4.2.2, we analyzed the lifetime of MPTCP connection with multiple interfaces, and then we proposed how to determine the number of interfaces needed to meet a required reliability.

#### 4.2.1. Analysis of Transmission Completion Time

The longer a UAV cannot communicate, the higher the probability that an accident will occur. Therefore, it is necessary to ensure a high rate of control packet transmission. *ConClone* uses multiple interfaces to increase the probability of successful transmission of control packets. Since MPTCP operates based on multiple interfaces, multiple connections can be established. When the number of interfaces is defined as *n*, a total of n−1 replicated control packets are generated. Replicated packets are transmitted simultaneously through each interface. In addition, if even one control packet is successfully transmitted, *ConClone* goes to the next process of transmitting the next control packet. If the probability of transmission failure in one process is assumed to be $f_{i}$ on average, the average successful transmission probability of system $P_{\mathrm{success}}$ can be calculated as follows:

(1) $$\begin{array}{cc} P_{\mathrm{success}} & =1-f_{1}\times f_{2}\times f_{3}\times \cdots \times f_{n}\\ \; & =1-\prod_{i=1}^{n}f_{i}. \end{array}$$

Equation (1) can be seen in most of parallel systems. MPTCP allows multiple interfaces to be used simultaneously. Assuming that the transmission failure probabilities of the each interface are the same as *f*, Equation (1) can be simplified as follows:

(2) $$P_{\mathrm{success}}\approx 1-f^{n}.$$

**Figure 3:**
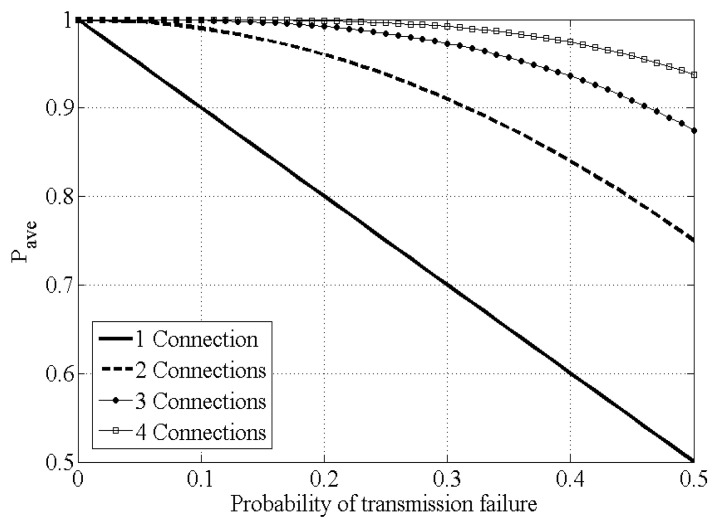
Successful transmission probability analysis.

The analysis result of the Equation (2) is shown in Figure 3. If the probability of transmission failure of single connection is 10%, the successful control packet transmission probability of system calculated through theoretical analysis is 0.900 when there is one connection; 0.990 when the connections number two with *ConClone*; and 0.999 when there are three connections with *ConClone*. As can be seen from those results, even if only two connections are used, most of the packet losses in ordinary network communication can be eliminated. Overall, it can be concluded that as the number of interfaces (*n*) increases, the successful transmission probability of system ($P_{\mathrm{success}}$) increases.

In order for control packets to be delivered to a UAV reliably, control packets must be transmitted through a reliable transmission protocol, TCP. The TCP maintains reliable transmission by retransmitting a packet that has failed to transmit. However, when retransmission occurs, the time for the control packet transmission to be completed (${\mathrm{Total}}_{\mathrm{completion}}$) is lengthened. Even if the transmission of control packet is reliable, it is difficult to maneuver the UAV moving at high speed if the transmission completion time of all control packets is not guaranteed. We theoretically analyzed how much *ConClone* increases the ${\mathrm{Total}}_{\mathrm{completion}}$ of the transmission compared to the ideal transmission time (${\mathrm{Total}}_{\mathrm{ideal}}$), and showed that it can ensure the stability of the UAV.

To see the effect of *ConClone* on the transmission completion time of transmitting *C* control packets, we first break down the time into total successful transmission time and total retransmission time. Each control packet takes several retransmissions until it is successfully transmitted, and its subsequent packet takes the same procedure. We initially derive the total transmission time ${\mathrm{Total}}_{\mathrm{ideal}}$ if there are no failures for each transmission. Note that this time is still present in other cases where a transmission failure appears, and in this case ${\mathrm{Total}}_{\mathrm{ideal}}$ is simply ${\mathrm{Total}}_{\mathrm{success}}$. Then we calculate total delay incurred by retransmissions, which is denoted by ${\mathrm{Total}}_{\mathrm{recovery}}$.

Firstly, we explain how to determine the total transmission time for whole *C* control packets. Let Ti denote packet transmission interval. Then, the ideal transmission time ${\mathrm{Total}}_{\mathrm{ideal}}$ for successful transmitting *C* control packets can be approximated as Equation (3):

(3) $${\mathrm{Total}}_{\mathrm{ideal}}\approx C\times Ti.$$

Secondly, we introduce a new packet transmission interval, which is different from Ti and occurs when one packet transmission is not successful. The new interval is larger than Ti because the transmission interval is determined by a TCP RTO, which directly notifies transmission failure. When any transmission failure occurs in TCP, TCP packet transmission is accompanied by failure detection (TCP RTO) and retransmission, both of which together are defined as *recovery* in this analysis. To derive $Ti_{\mathrm{recovery}}$, which is the aggregate retransmission time to transmit *C* control packets, we define one failed transmission interval ($Ti_{\mathrm{failure}}$) with failure coefficient *k*, expressed as follows:

(4) $$Ti_{\mathrm{failure}}\approx k\times Ti,\;\left(k>1\right).$$

In the above equation, the coefficient *k* is the failure coefficient that expresses the extent to which the transmission time is lengthened due to the transmission failure.

Thirdly, we derive the aforementioned $Ti_{\mathrm{recovery}}$. Since a failed packet transmission can cause several TCP retransmissions until the successful packet transmission, we define the recovery time for one packet ($Ti_{\mathrm{recovery}}$) as the time taken for those retransmissions. In the event of a packet transmission failure, the probability of failure for a single transmission is obtained as system failure probability of $1-P_{\mathrm{success}}=f^{n}$ from Equation (2). Therefore, the failure probability of j−th for one packet is ${\left(f^{n}\right)}^{j}$. Since, $Ti_{\mathrm{recovery}}$ should be defined for infinite TCP retransmissions, from $Ti_{\mathrm{failure}}$ in Equation (4) and ${\left(f^{n}\right)}^{j}$, $Ti_{\mathrm{recovery}}$ is expressed as follows:

(5) $$\begin{array}{cc} Ti_{\mathrm{recovery}} & =Ti_{\mathrm{failure}}\left(1+f^{n}+f^{2n}+\cdots \right)\\ \; & =k\times Ti\times \left(\frac{1}{1-f^{n}}\right). \end{array}$$

Then, ${\mathrm{Total}}_{\mathrm{recovery}}$ is expressed as the sum of the total recovery time for *C* packet transmissions. The probability that the system fails to transmit for the first time, $1-P_{\mathrm{success}}$, is reflected in $Ti_{\mathrm{recovery}}$, so ${\mathrm{Total}}_{\mathrm{recovery}}$ is as follows:

(6) $$\begin{array}{cc} {\mathrm{Total}}_{\mathrm{recovery}} & \approx C\times \left(1-P_{\mathrm{success}}\right)\times Ti_{\mathrm{recovery}}\\ \; & =C\times f^{n}\times k\times Ti\times \left(\frac{1}{1-f^{n}}\right). \end{array}$$

Finally, we derive the ratio of completion time to ideal time when there are retransmissions. The total time spent just on successful transmissions for *C* packets, ${\mathrm{Total}}_{\mathrm{success}}$, is equal to ${\mathrm{Total}}_{\mathrm{ideal}}$, which is the time for ideal transmissions without any failure. By adding the transmission time (${\mathrm{Total}}_{\mathrm{success}}$) to the required retransmission time (${\mathrm{Total}}_{\mathrm{recovery}}$), the time to make all transmissions completed (${\mathrm{Total}}_{\mathrm{completion}}$) can be calculated. With Equations (3) and (6), the rate of increase in completion time (Tr) can be thus calculated as:

(7) $$\begin{array}{cc} Tr & =\frac{{\mathrm{Total}}_{\mathrm{completion}}}{Tota{\mathrm{Total}}_{\mathrm{ideal}}}=\frac{{\mathrm{Total}}_{\mathrm{success}}+{\mathrm{Total}}_{\mathrm{recovery}}}{{\mathrm{Total}}_{\mathrm{ideal}}}\\ \; & \approx \frac{C\times Ti+C\times f^{n}\times k\times Ti\times \left(\frac{1}{1-f^{n}}\right)}{C\times Ti}\\ \; & =1+\frac{k\times f^{n}}{\left(1-f^{n}\right)}. \end{array}$$

**Figure 4:**
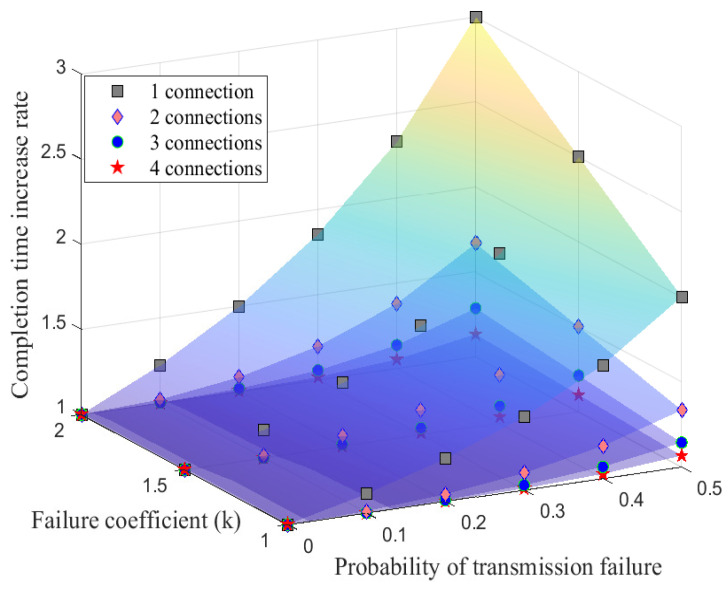
Completion time analysis.

Figure 4 shows that the completion time increase ratio (Tr) decreases as the number of connections increases with failure coefficient *k* varies over 1 and 2.

In the case of k=1.5, if UAV control requires the delay of the control packet to be 10% or less, it can be expressed as Tr<1.1. In the case of the existing MPTCP, in order to satisfy Tr<1.1, it can be achieved only when the probability of transmission failure of connection (*f*) is 9% or less. However, when control packets are transmitted to two connections with *ConClone*, Tr<1.1 is satisfied even when the probability of transmission failure is 29% and the probability is a reasonable condition that is analyzed at a distance of around 80 m (which will be discussed in Section 4.3). With three connections, the probability of transmission failure up to 44% can be tolerated. Overall, it is possible to reliably transmit control packets with *ConClone* even in a high failure environment.

#### 4.2.2. Number of Interfaces Required to Ensure Expected Reliability

**Figure 5:**
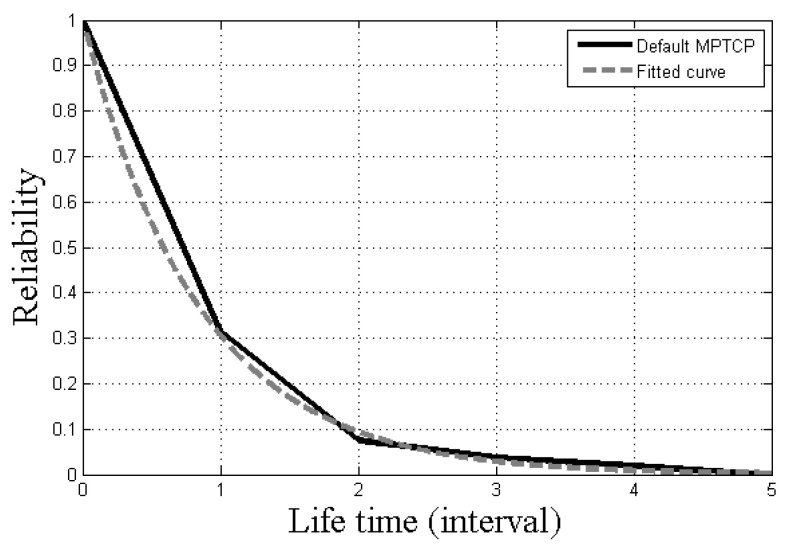
The reliability of the lifetime interval and its fitting results.

We experimented on empirical packet transmission based on MPTCP. As described in Section 5, the UAV and ground control system (GCS) communicated with each other by one hop. GCS transmitted control packets at regular intervals. We measured the inter-packet time of control packets, and the results are shown in Figure 5.

As shown in Figure 5, we found that the lifetime interval of MPTCP transmission can be approximated with an exponential distribution. In the exponential distribution with parameter λ, the expectation is 1/λ.

**Figure 6:**
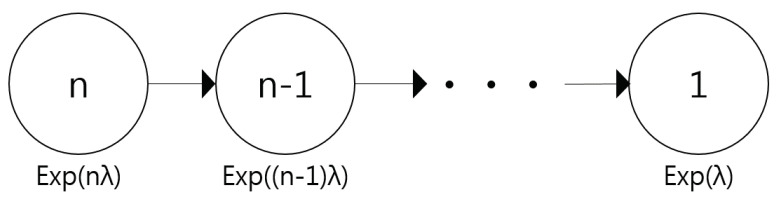
The lifetime distribution of *ConClone*.

The lifetime interval indicates the number of successes in transmitting control packets continuously without retransmission. As can be seen in Figure 6, *ConClone* works when only one of *n* interfaces is active, so the lifetime of *ConClone* can be modeled as a series of intervals where each interval *i* is the interval in which *i* number of interfaces are active. Therefore, the distribution of *ConClone*’s lifetime can be modeled with *n*-stage serial system, where the lifetime of each stage *i* is also exponentially distributed with parameter iλ, denoted as $X_{i}∼EXP(i\lambda$).

If there are *n* interfaces, the entire process until the number of inactive interfaces increases, leaving one available interface being defined as the lifetime of *ConClone*. Then the lifetime of *ConClone*, *Y* can be defined as follows:

(8) $$\begin{array}{cc} Y & =X_{n}+X_{n-1}+\cdots +X_{1}. \end{array}$$

Therefore, the expected reliability of the entire system (E[Y]) is calculated as follows:

(9) $$\begin{array}{cc} E[Y] & =E[X_{n}+X_{n-1}+\cdots +X_{1}]\\ \; & =E\left[X_{n}\right]+E\left[X_{n-1}\right]+\cdots +E\left[X_{1}\right]\\ \; & =\frac{1}{n\lambda }+\frac{1}{(n-1)\lambda }+\cdots +\frac{1}{\lambda }\\ \; & =\frac{1}{\lambda }\times \left(\frac{1}{n}+\frac{1}{n-1}+\cdots +1\right)\\ \; & =\frac{1}{\lambda }\times H_{n}\\ \; & \approx \frac{1}{\lambda }\ln\left(n\right). \end{array}$$

Assume the required expected reliability (MTTF) is given with at least α; E[Y] must be greater than or equal to α. With Equation (9), the number of interfaces *n* required to ensure the expected reliability α can be expressed as follows:

(10) $$\begin{array}{cc} n & =e^{\lambda \alpha }. \end{array}$$

**Figure 7:**
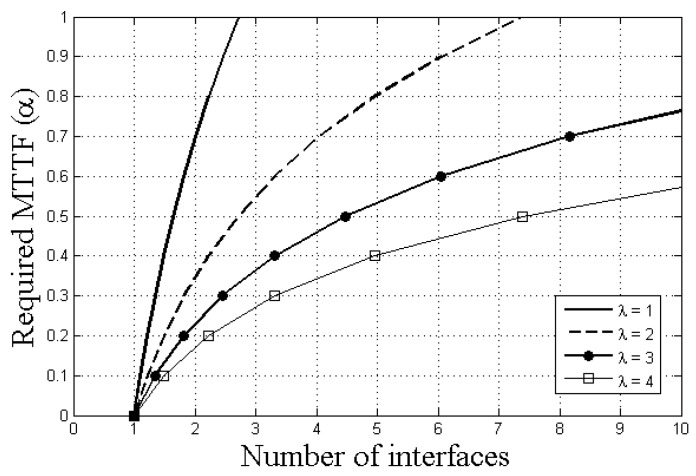
Number of interfaces required to ensure expected reliability.

Equation (10) is plotted as shown in Figure 7. For example, if the λ is 2 and the required reliability (α) is 0.6, at least n=4 interfaces are required to ensure reliability. If the required reliability rises to 0.8, five interfaces are necessary. If the λ is reduced to 1 when the required reliability is still 0.8, the number of required interfaces is reduced to three. Overall, through Equation (10), we can understand how many interfaces are required to ensure the required reliability. Note that this explanation is only applied to the network configuration specified in the initial part of this analysis. As for other configurations for network parameters, such as deadline and network delay, we need to figure out the λ and then determine the necessary number of interfaces for the required reliability.

### 4.3. UAVs Channel Model Analysis

In this subsection we analyzed on TCP packet transmission failure based on path loss (PL) that reflected the effect of the channel model for real UAV operation. Aerial link characterizations in both air-to-ground (A2G) and air-to-air (A2A) situations were sufficiently analyzed in [37], using 802.11 interfaces, and the long-distance pass loss model is shown in Equation (11):

(11) $$PL=PL\left(d_{0}\right)+10\alpha \times \log_{10}\left(\frac{d}{d_{0}}\right)$$

where $d_{0}$ and *d* represent the minimum distance and the distance between transmitter and receiver respectively. Channel analysis was performed with a configuration with $d_{0}$ of 1 m and *d* of 1 to 100 m based on this equation. According to [37], A2G and A2A channels have path loss exponents α as 2.03 and 2.01 respectively.

**Figure 8:**
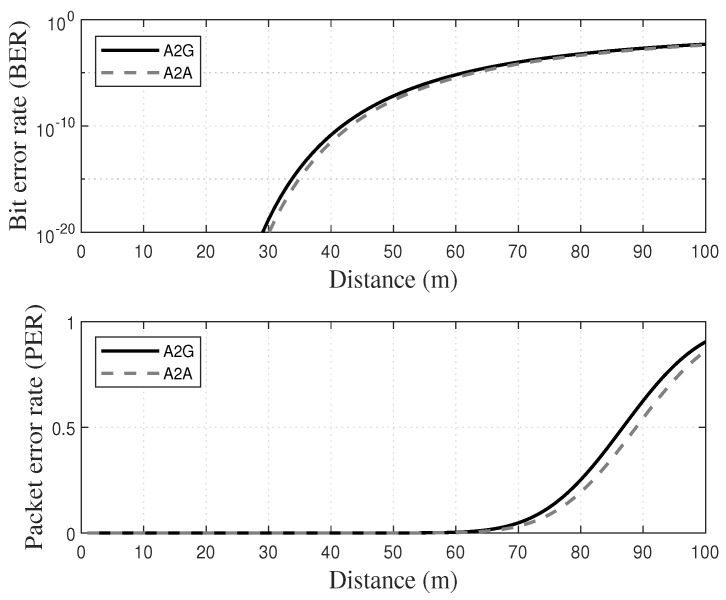
Bit error rate (BER) and PER based on the unmanned aerial vehicle (UAV) channel model.

With 802.11 g using *M*-order quadrature amplitude modulation (QAM), including constellation and code-specific constants $\kappa_{1}$ and $\kappa_{2}$, channel gain *h* from the PL, transmission power $p_{tr}$ and bit error rate (BER) are approximated as Equation (12), which is in [38]. Furthermore, in the communications with the frame length $L_{fr}$, the frame error $f_{\mathrm{frame}}$, by some error of bits within a single frame, is calculated as follows:

(12) $$BER=\kappa_{1}\times \exp\left(-\frac{\kappa_{2}hp_{tr}}{2^{\rho }-1}\right),$$

(13) $$f_{\mathrm{frame}}=1-{\left(1-BER\right)}^{L_{fr}},$$

where $L_{fr}$ is the number of bits for a single frame. Packet error occurs in the case that all transmitted frames, including retransmissions, are not successful. Therefore the packet error probability can be obtained from the Equations (12) and (13). Additionally, we can approximate the packet loss probability $f_{\mathrm{packet}}$ in Equation (14)

(14) $$f_{\mathrm{packet}}=1-{\left(1-f_{\mathrm{frame}}^{N_{re}+1}\right)}^{N_{fr}},$$

where $N_{fr}$ is the number of frames within a single packet and $N_{re}$ is the number of frames retransmitted within the single packet. The derived $f_{\mathrm{packet}}$ is planned to solidify the analysis and experiment with *ConClone* shown in Figure 8. Furthermore, the setting of *f* in the theoretical derivation of Section 4.4 and empirical experiments of Section 5 is based on this analysis.

### 4.4. Hardware Stability Analysis

Enhancing the stability of UAVs from the additional network interfaces can be done by analyzing the control system with the Newton–Euler theorem. We use fundamental values of UAVs in the case of a quad-copter to improve the stability. These values are the rotor’s angular velocity (*w*), air density generated by the propeller (*B*), torque (τ), axis (*T*) and the distance form the center of mass to the rotor (*L*). In our case of a quad-copter, quadrants are defined as A1, B1, A2 and B2 clockwise. According to [39], the variables of the inertia moments I of the quad-copter for the x-axis (pitch) and y-axis (roll) and its thrust to attitude as z-axis (yaw) are described in Equation (15):

(15) $$I=\left[\begin{array}{c} I_{xx}\\ I_{yy}\\ I_{zz} \end{array}\right]=\left[\begin{array}{c} \frac{2}{5}M_{T}R_{C}^{2}+\left(I_{A1}+I_{A2}\right)\\ \frac{2}{5}M_{T}R_{C}^{2}+\left(I_{B1}+I_{B2}\right)\\ \frac{2}{5}M_{T}R_{C}^{2}+\left(I_{A1}+I_{A2}+I_{B1}+I_{B2}\right) \end{array}\right]=\left[\begin{array}{c} \frac{2}{5}M_{T}R_{C}^{2}+2L^{2}m_{r}\\ \frac{2}{5}M_{T}R_{C}^{2}+2L^{2}m_{r}\\ \frac{2}{5}M_{T}R_{C}^{2}+4L^{2}m_{r} \end{array}\right].$$

On a single axis, the *y*-axis as in our case, an additional pair of network interfaces can be mounted near each rotor. Therefore, changed moments of inertia matrix $I^{{}^{\prime }}$ are rewritten with the mass $m_{i}$ of *n* interfaces as Equation (16).

(16) $$I^{{}^{\prime }}=\left[\begin{array}{c} I_{xx}^{{}^{\prime }}\\ I_{yy}^{{}^{\prime }}\\ I_{zz}^{{}^{\prime }} \end{array}\right]=\left[\begin{array}{c} \frac{2}{5}\left\{M_{T}+\left(n-1\right)m_{i}\right\}R_{C}^{2}+2L^{2}\left(m_{r}+\lfloor \frac{n}{4}\rfloor m_{i}\right)\\ \frac{2}{5}\left\{M_{T}+\left(n-1\right)m_{i}\right\}R_{C}^{2}+2L^{2}\left(m_{r}+\lfloor \frac{n+2}{4}\rfloor m_{i}\right)\\ \frac{2}{5}\left\{M_{T}+\left(n-1\right)m_{i}\right\}R_{C}^{2}+4L^{2}\left(m_{r}+\lfloor \frac{n}{2}\rfloor m_{i}\right) \end{array}\right],\left(n=1,2,3,\cdots \right).$$

Our quad-copter with the DJI F450 frame has a 0.25 m distance *L*, a 0.07 m radius, a 1013 g total mass and a 16 g interface. Furthermore, as shown in Equation (16), we assumed the additional network interfaces were adapted in a symmetrical position. Therefore, symmetrically mounted interfaces on both ends of each frame increased the mass of motor parts ($m_{r}$) and total mass ($M_{T}$) slightly. There was no alteration to the center of mass. With these values, we derived how the inertia moments of the quad-copter are changed for the cases with 1 to 4 interfaces in Table 1. The minimum value was 0.0057 for $I_{xx}$ and $I_{yy}$ of the single interface and the maximum value was 0.0176 $I_{zz}$ for the four interfaces. The moment of inertia determines how agile and reliably control units such as Pixhawk can control the UAV. The changed moment of inertia was small enough to compare with the conditions of several studies of UAV control stability with specifications similar to our quad-copter [40,41,42,43].

## 5. Evaluation

This section describes experiments and results to demonstrate the performance of *ConClone*. The *ConClone* implementation is described first. Then we describe the experiment and the results using real devices. Finally we succinctly describe the specific application scenarios using *ConClone*.

### 5.1. Implementation and Configuration for Evaluation

To run *ConClone* on a portable computing board that can be mounted on UAV, we implemented *ConClone* on Ubuntu 16.04 LTS, an operating system that can be used on a portable computing board [44]. *ConClone* was developed based on MPTCP v0.91.3. To use MPTCP and sk_buff as described in Section 3.3.1, we implemented *ConClone* in the kernel layer of Linux. In the experiment, two computing boards with *ConClone* were used, and each computing board was equipped with two low-cost IEEE 802.11g network interfaces. Therefore, computing boards could communicate simultaneously with two connections. In the experiment, one network interface antenna was removed to produce the network environment degradation in which control packets were lost.

The Pixhawk 4 autopilot used to control the UAV was configured to periodically receive control packets within 0.5 s to reliably control the UAV. In addition, during real UAV flight, various information other than control packets was exchanged between the GCS and the computing board of UAV. Reflecting this, when the control packet was delivered, the next control packet was sent at 0.5 s intervals and the general data packet was transmitted at the same time. We experimented until 120 control packets were delivered and compared the performance between *ConClone* and MPTCP using the default scheduler.

### 5.2. Evaluation of Inter-Control Packet Delay

**Figure 9:**
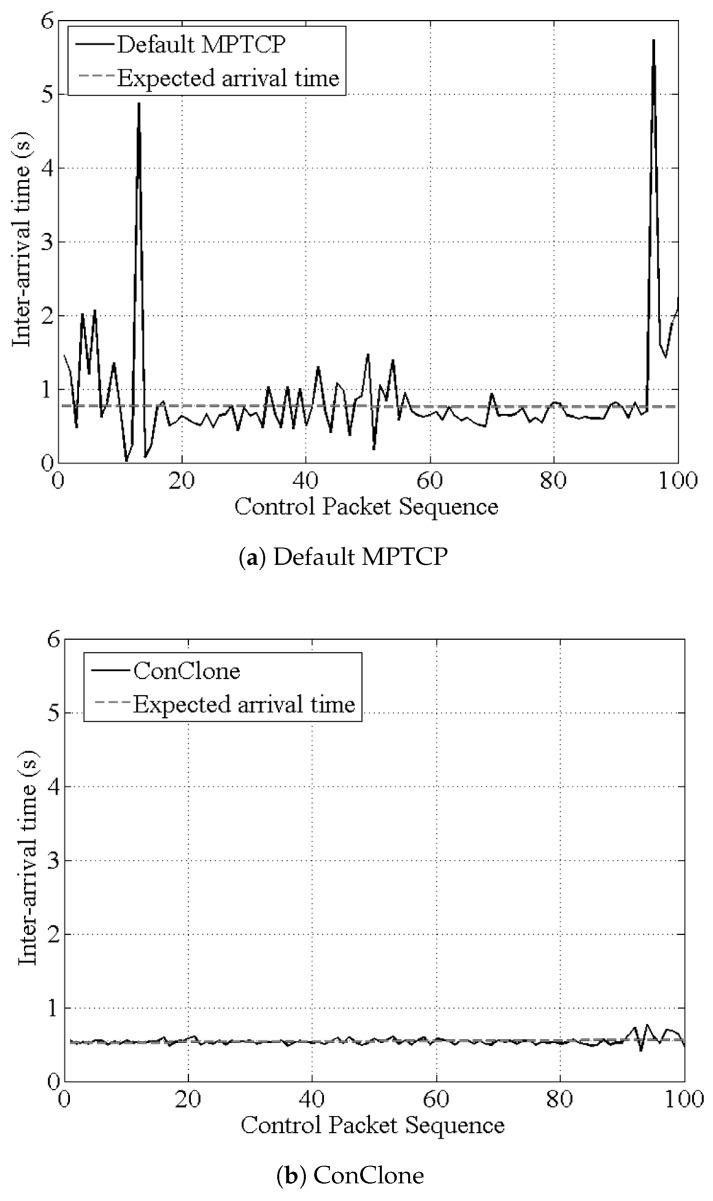
Comparison with expected inter-packet time.

Figure 9 shows the comparison with the estimated inter-packet time calculated by Equation (7). As shown in Figure 9a, the default MPTCP had increased transmission time due to retransmission, and showed a significant deviation from the expected inter-packet time. On the other hand, *ConClone* showed results similar to the expected inter-packet time, and was very stable.

**Figure 10:**
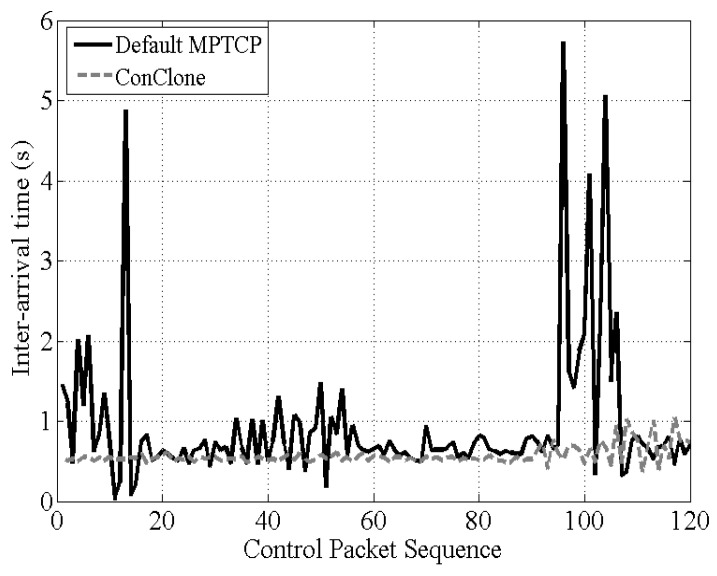
Inter-packet times of control packets.

**Figure 11:**
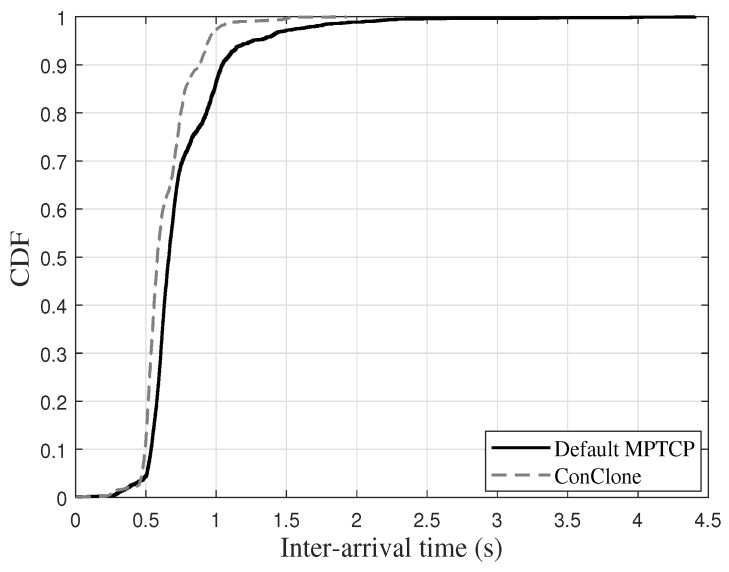
Cumulative distribution function (CDF) of inter-arrival time in long-term transmission.

Figure 10 shows the inter-packet time between control packets measured by the receiver. MPTCP using the default scheduler showed irregular inter-packet time. However, *ConClone* showed stable inter-packet time close to 0.5 s. When a control packet loss occurred, the lost control packet was retransmitted again, and the inter-packet time was increased by the delay due to the retransmission. Therefore, the observed stability of inter-packet time in *ConClone* indicated that *ConClone* rarely could cause packet loss. The averages and standard deviations of the results obtained in the experiment of Figure 10 are shown in Table 2. *ConClone* was close to the transmission interval of the control packet; the standard deviation was much less than the default MPTCP. Additionally, *ConClone* showed stable communication even in long-term transmission of more than 2000 control packets, which is shown in Figure 11. The inter-arrival time for 13.6% of packets transmitted based on Default MPTCP was more than 1 s, whereas only 2.8% of *ConClone* took more than 1 s. Moreover, the maximum inter-arrival time of *ConClone* was only 1.92 s. As a result, *ConClone* was able to show stable inter-packet time due to low control packet loss.

### 5.3. Evaluation on Transmission Completion Time

As in the experiment in Section 5.2, when the control packet was delivered, the next control packet was transmitted after 0.5 s. Therefore, all transmissions should have ended in 120×0.5 s. However, if a control packet was lost and retransmission occurred, it would be delayed, so the completion time of transmitting 120 control packets became longer.

**Figure 12:**
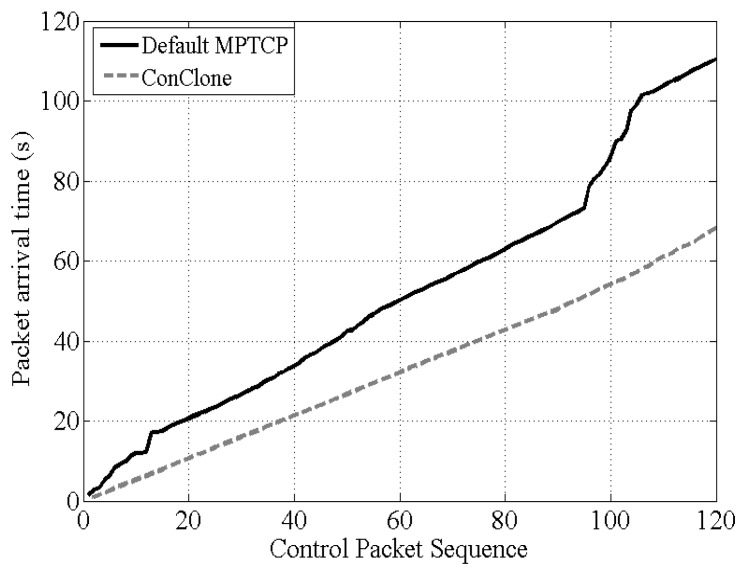
Completion times of all control packet transmissions.

Figure 12 shows the time taken for all control packets to be delivered. Default MPTCP delivered 120 control packets for a total of 110.44 s, but *ConClone* transmitted all in 68.17 s. Unlike the default MPTCP, the *ConClone* was unaffected by retransmission, so *ConClone* took less time to transmit 120 packets than the default MPTCP. This result can also be seen as the theoretical completion time calculated from Equation (7). In this experiment, packet loss occurred on average with 28.4% probability. When the error rate was 28.4%, the theoretical completion time increase rate was 1.5540 for one connection and 1.0954 for two connections. Since the default MPTCP transmitted only one control packet at a time, it could be considered that only one connection was actually used to transmit the control packet. However, since *ConClone* replicated the control packet and transmitted through all connections, it could secure the transmission rate corresponding to two connections.

The theoretical time required to transmit all 120 packets is 86.702 s for default MPTCP and 65.906 s for *ConClone* for failure coefficient k=1.122 in this evaluation. Table 3 summarizes these results. Table 3 shows that the theoretical and experimental results of *ConClone* were similar to each other. In real experiments, conventional MPTCP could not cope with transmission failure due to congestion and connection instability. Therefore, the time required for the transmission of all control packets was longer than that of *ConClone*. Retransmission was considered in the theoretical model, but the temporary instability of the connection in UAV was not considered. Thus, the difference between the actual experiment and the theoretical time was as much as the time until the connection was restored. As future work, we will revise the theoretical model to include connection instability time. Overall, unlike conventional TCP or MPTCP, control packets can be transmitted reliably through *ConClone*, while maintaining the MPTCP default scheduler, which can achieve high throughput with multiple connections.

### 5.4. Real Scenarios

We envision that *ConClone* can be utilized in many real scenarios where reliable and time-constrained communication is required for applications with UAVs. First, in the works for UAV gas sensing, it is necessary to accurately update the measured gas distribution map through real-time networking [45,46,47]. With *ConClone*, we can shorten the transfer completion time of sensing and localization data, thereby ensuring stable transfer of that data. In particular, Kersnovski et al. proposed a solution in terms of the exploration algorithm in a multi-UAV framework to avoid mission failure in a real world scenario [48]. Through the application of *ConClone*, it is possible to reduce the failure rate or to quickly recover the connection in this real scenario. In addition, several studies have been proposed in [49,50] in terms of routing paths and physical links to ensure strong connectivity in A2G and A2A. At the transport level, the proposed *ConClone* can be effectively applied with little or no dependency between link and routing, and can construct robust UAV connections.

## 6. Conclusions

UAVs have high maneuverability that is not obstructed by terrain and objects. Considering the advantages of UAVs, many researchers are carrying out studies to apply them to various tasks. However, because UAVs are flying at a high speed, it is possible that an accident may occur if the control packet transmission is delayed for a moment. Considering the weight of a UAV and its rotating rotor blades, a collision with any UAV would be very dangerous. Therefore, in order to prevent accidents, the control packet must be stably transmitted to the UAV. To ensure stable UAV control, we proposed *ConClone*. *ConClone* replicates control packets and transmits them through multiple connections to increase the probability of successful delivery of control packets and minimize delay. Additionally, we proved the performance of *ConClone* theoretically and proved the stability of the *ConClone* by conducting experiment with real devices.

As future work, we would like to strengthen *ConClone* by understanding the situation wherein packet replication occurs or by analyzing the replication frequency through deep learning. We also plan to modify the modeling to account for long-term transmission instability. If the modeling can take into account the transmission instability that makes retransmissions impossible, then the time required for transmission can be more accurately estimated. Moreover, further rigorous research into the cross-layer approach to MPTCP [51,52] and channel modeling [53] has the potential to significantly improve performance when applying *Conclone*. Therefore, we will perform additional cross-layer analysis as future work that considers MPTCP, multiple interfaces, channel characteristics and their mutual influences, and then apply the results to *ConClone*.

## Figures and Tables

**Table 1:** Moments of inertia according to the number of additional interfaces.

Moments of Inertia	Single Interface	2 Interfaces	3 Interfaces	4 Interfaces
$I_{xx}$(kg × m${}^{2}$)	0.0057	0.0058	0.0058	0.0078
$I_{yy}$(kg × m${}^{2}$)	0.0057	0.0078	0.0078	0.0078
$I_{zz}$(kg × m${}^{2}$)	0.0095	0.0135	0.0135	0.0176

**Table 2:** Average and standard deviation of experiment with real devices.

Scheme	Average (s)	Std
Default MPTCP	0.9204	0.8637
*ConClone*	0.5681	0.1135

**Table 3:** A comparison of real and theoretical completion times.

Scheme	Real (s)	Theoretical (s)
Default MPTCP	110.44	86.702
*ConClone*	68.17	65.906

## Data Availability

Data sharing not applicable.

## Abbreviations

The following abbreviations are used in this manuscript:

UAV	Unmanned Aerial Vehicle
MPTCP	MultiPath Transmission Control Protocol
